# Correlation between Kind of Cesarean Section and Posttraumatic Stress Disorder in Greek Women

**DOI:** 10.3390/ijerph17051592

**Published:** 2020-03-01

**Authors:** Eirini Orovou, Maria Dagla, Georgios Iatrakis, Aikaterini Lykeridou, Chara Tzavara, Evangelia Antoniou

**Affiliations:** 1 Department of Midwifery, University of West Attica, 12243 Athens, Greece; mariadagla@uniwa.gr (M.D.); giatrakis@uniwa.gr (G.I.); klyker@uniwa.gr (A.L.); lilanton@uniwa.gr (E.A.); 2 National and Kapodistrian University of Athens, Faculty of Medicine, 15784 Athens, Greece; htzavara@med.uoa.gr

**Keywords:** posttraumatic stress disorder, PTSD Profile, emergency cesarean section, elective cesarean section

## Abstract

A birth experience with cesarean section (CS) can be a cause of the development of post-traumatic stress disorder after a cesarean (PTSD-AC) or profile PTSD, for a percentage of women. So far, there is no data on the frequency of PTSD-AC in Greece and this syndrome is often associated with other mental disorders of the postpartum period. The purpose of this research is to associate the kind of CS with PTSD-AC for Greek mothers and the combination of factors that make them less resistant to trauma. A sample of ahundred and sixty-six mothers who gave birth with emergency cesarean section (EMCS) and elective cesarean section (ELCS) at a Greek University hospital have consented to participate in the two phases of the survey, in the 2nd day postpartum and a follow-up in the 6th week postpartum. Medical/demographic data and a life events checklist (LEC-5) with Criterion A and post-traumatic stress checklist (PCL-5) were used to diagnose PTSD and PTSD Profile. Out of166 mothers enrolled, 160 replied to the follow-up (96.4%), ELCS 97 (97%) and EMCS 63 (95%). Twenty (31.7%) EMCS had PTSD and nine (14.3%) had Profile. One (1%) ELCS had PTSD and 4 (4.1%) had Profile. This survey shows a high prevalence rate of PTSD after EMCS with additional risk factors of preterm labor, inclusion in the Neonatal Intensive Care Unit (NICU), a lack of breastfeeding, and a lack of support from the partner.

## 1. Introduction

Posttraumatic stress disorder(PTSD) is a trauma-related stress disorder caused by exposure to real or threatened death, heavy injury or a threat of physical integrity in various forms of exposure, such as direct exposure, exposure as a witness, exposure through information or extreme repeated exposure to the workplace. The disorder, regardless of the type of exposure to trauma, causes symptoms of re-experiencing, avoidance, negative cognitions in the mood and arousal. The disturbance lasts more than a month, not due to the action of any substance or physical condition and causes a significant reduction in the person’s social life [1]. The prevalence of PTSD is two times greater in women than in men whichshows how it is influenced by childbirth experiences, hormonal disorders, stressful life events and domestic violence [2].

On the other hand, PTSD Profile includes the most important symptoms of PTSD, yet people exposed to the trauma, do not meet all the diagnostic criteria of the disorder. This is partial PTSD, which has been shown to be associated with increased rates of alcoholism, suicidal ideation, overconsumption of health services, and an increase in absences in the working environment.

Additionally, a significant reduction in the social and professional life of the individual is observed [3,4].

### 1.1. PTSD after Childbirth

For several years, the experience of the birth of a child was viewed by scientists as a positive experience for the mother. In recent years, however, research into women with traumatic birth experiences has increased interest and it is now known that some of these experiences may lead to trauma disturbances. More than 1/3 of women experience birth as a traumatic event [5], while approximately 1/4 of them will experience postpartum PTSD [6].

The perinatal period is special for every woman’s life. Most of them experience birth as a pleasant experience, but in a small percentage, this experience becomes a traumatic event, affecting to a significant extent the woman herself and everyone around her. Various conditions seem to affect the development of this disorder, such as pregnancy pathology, complications during birth, emergency cesarean section, history of psychiatric disorders, fear during childbirth, and previous traumatic events in the mother’s life [7,8,9,10]. A traumatic birth experience that evolves into PTSD or PTSD Profile can overshadow the mother-child relationship, the relationship with the partner, and the desire to acquire another child in the future [11]. When there are past traumatic events in the mother’s life, it is very difficult to determine what has caused the symptoms of PTSD postpartum. After a birth experience, it is known that past traumas of the mother’s life can be recalled and cause symptoms that lead to the re-experiencing of an old PTSD [7,8,12].

### 1.2. PTSD after Cesarean(AC)

Many researchers have been carried out on the effect of the kind of delivery on the development of PTSD postpartum [11,13,14,15]. Regarding cesarean sections (CS), however, there are surveys that do not differentiate the effect between emergency cesarean section (EMCS) and elective cesarean section (ELCS) and they finally considered that there is no correlation betweenEMCS and PTSD [16,17,18,19]. In contrast, however, both old and new surveys show a great correlation between EMCS and PTSD compared to other types of birth [6,12,20], while only two investigate the correlation of EMCS with PTSD [21,22]. Some researchers also consider that the psychosocial characteristics of women, previous traumas, and history of mental disorders are stronger risk factors than EMCS in the development of PTSD [8,23,24].

### 1.3. PTSD- AC in Greek Women

Every year in Greece, there are about 90,000 births [25] of which more than half are CS. Although the WHO recommends that the rates of CS do not exceed 10-15% of births, Greece is in the highest position worldwide [26], which makes Greek women more exposed to birthtrauma [27]. So far, there is no research study of PTSD disorders in Greek women, while the data is limited to other mental disorders of the postpartum period [28,29]. The purpose of this investigation is first to study the frequency of PTSD between two groups of women—EMCS and ELCS—in the 6th week postpartum and secondly, to determinethe risk factors and their degree of contribution to the development of specific postpartum disorders. It is expected after identification of the risk factors, to develop specialized obstetric interventions of prevention and treatment of PTSD after cesareans(AC) in the future.

## 2. Materials and Methods

This prospective study took place from July to November 2019, at the obstetrics clinic of the General University Hospital of Larissa in Greece. It was approved by the University Hospital of Larisa Ethics Commission. Approval: 18838/08-05-2019.The present study is using a descriptive design for the recording of the prevalence of PTSD-AC within 6 weeks postpartum and the risk factors that may lead to the development of this disorder.

### 2.1. Participants

Survey participants wereall the women who gave birth with EMCS or ELCS and gave their written consent for their participation. All women had a medical surveillance dossier from which the demographics and medical data were obtained. Excluded from the research were the women with difficulties in understanding the Greek language or other difficulties at a cognitive level which would create a problem in understanding the questions or the measuring tools. Additionally, the underage mothers were excluded, or those who used psychotropic substances or drugs and all those who gave birth with EMCS from other hospitals in the region.

### 2.2. Data and Measures

The data was collected in 2 stages: the first stage wasthe 2nd day afterchildbirth, and; thesecondstage wasthe 6th week after childbirth. During the firststage that coincides with the recovery of the woman after surgery, from the 160 women who met the criteria for participation, medical and demographic data were collected, old traumatic life events and the identification or not of theCS being a traumatic experience. Inthe second stage, through a telephone interview, respondents answered questions on the PTSD scale. The specific period of time was selected in order to meet the criterion of the duration of symptoms over 4 weeks [30].

All measures were authored by the National Center of PTSD Staff according to the Diagnostic and Statistical Manual (DSM V), and weretranslated and weighted into the Greek language by the investigator midwife.

#### 2.2.1. Socio-Demographic Questionnaire

The research-made screening form includes items on demographic, social, medical (obstetric- neonatal) and mental characteristics of the participants. Italso included information on the experience of the traumatic cesarean section.

#### 2.2.2. Life Events Checklist-5 (LEC-5) of DSM-V

This measure of exposure to traumatic events of the past is used with PCL-5 to determine exposure to a traumatic event [31]. The life events checklist (LEC) is the only screening tool that respondents can determine different levels of exposure to a traumatic event in their lives, while it appears to have adequate psychometric properties similar to other measures of traumatic life events [32].

#### 2.2.3. Criterion A of DSM-V

To confirma PTSD diagnosis, 8 criteria must be met. For Criterion A, the person must have been exposed to death, threatened death, serious injury or sexual violence in one of the following ways: (a) direct exposure, (b) witness to the event, (c) information of the event, and (d) exposure in the working space [33,34]. For the purposes of this investigation, which concerns direct exposure of women to a traumatic birth experience, criterion A has been adapted with appropriate questions that determine the exposure of the mother or baby to death or threatened death or serious health complications of both according to the requirements of the DSM-V.

#### 2.2.4. Post-Traumatic Stress Checklist (PCL-5)of DSM-V

The post-traumatic stress checklist (PCL-5) is a self-report measure, which was developed to measure and evaluate PTSD and PTSD Profile symptoms. In the present investigation, the respondents replied via telephone to 20 questions, corresponding to 20 symptoms of the criteria B (re-experiencing), C (avoidance), D (negative thoughts and feelings), and E (arousal and reactivity). All replies are scored by 5-point scales (range zero to four). A score of one or more in the categories of criteria B and C and two or more in categories D and E are considered clinical findings. If all 4 criteria are met in connection with criterion A, the diagnosis of PTSD-AC is considered possible. To determine the severity of the symptoms, the method of the sum of the score of all responses with a PCL score ≥ 33was also used [35]. Depending on the symptoms, the diseased, separated into (a) provisional diagnosis of PTSD and (b) PTSD Profile (criterion B, C, D) [3,4,19]. PCL-5 is a measure with very good psychometric properties for diagnosing PTSD symptoms in all population groups [36,37].

### 2.3. Statistical Analysis

Quantitative variables were expressed as mean values (SD) or as median values (interquartile range = IQR). Qualitative variables are presented with absolute and relative frequencies. For the comparisons of proportions, chi-square and Fisher’s exact tests were used. Student’s *t*-tests were computed for the comparison of mean values when the distribution was normal and the Mann-Whitney test was used for the comparison of median values when the distribution was not normal. Logistic regression analyses were performed in order to identify factors associated with the presence of PTSD.Unadjusted and adjusted odds ratios with 95% confidence intervals were computed from the results of the logistic regression analyses. Statistical significance was set at 0.05 and analyses were conducted using SPSS statistical software (SPSS Statistics version 22.0, IBM, Armonk, NY, USA).

## 3. Results

Data from 160 women with a mean age of 33.1 years (SD=5.9 years) were analyzed. Sample characteristics in totals according to the type of cesarean section are presented in Table 1. Sixty-three (39.4%) of the cases had an urgent cesarean section and 97(60.6%) had a cesarean section. Age, family status, financial status, medical history, and nationality were similar in the two study groups. Women with a planned cesarean section were more likely to have had a previous birth or a previous cesarean section and less likely to have a psychiatric history. Additionally, the median number of traumatic events that wererecorded was greater in the group of women who hadan emergency caesarean section.

### 3.1. Pregnancy and Delivery Characteristics

**Figure 1:**
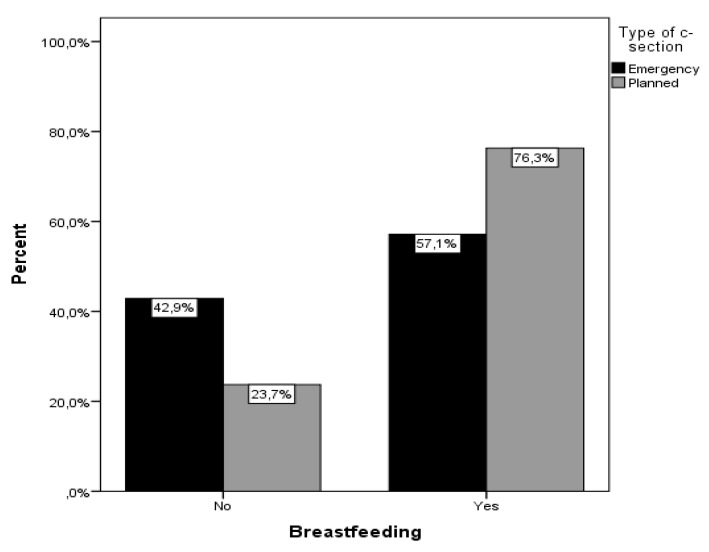
Type of cesarean section(CS) and rates of breastfeeding.

Pregnancy and delivery characteristics are shown in Table 2. In the emergency cesarean section group, a greater proportion of women had problems during pregnancy or required inclusion to the Neonatal Intensive Care Unit (NICU). Women with a planned cesarean section reported more support from their spouses and had significantly lower proportions of reported traumatic cesarean section. Furthermore, women with emergency cesarean section had a lower rate of breastfeeding (Figure 1) and expectations.

### 3.2. ΤhePrevalence of PTSD Criteria among Women after CS

**Figure 2:**
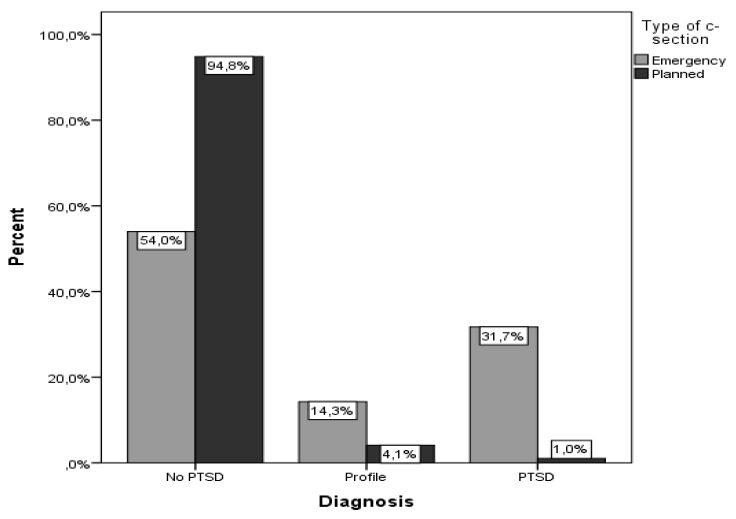
Proportion of subjects with posttraumatic stress disorder (PTSD) according to type of cesarean section.

The proportion of women with a profile of PTSD was 14.3% in the emergency cesarean section group and 4.1% in the planned cesarean section group, while the corresponding proportions for having PTSD were 31.7% and 1% (*p* < 0.001-Figure 2).

Table 3 gives an overview of PTSD and PTSD Profilecriteria in women after CS. Among the participants (*n* = 160), the proportion who met criterion A is 25.6% (EMCS 54% and ELCS 7.2%) and those who metcriterion B through to E, is between 19-26.3% (EMCS 36.4–52.5% and ELCS 7.2–10.3%). A total of 13, 1% (*n* = 21) of women after CS met all criteria (A, B, C, D, E) according to the DSM V, while 8.1% (*n*= 13) fulfilled criteria (B, C, D) and missed criteria A and E.

Table 3 Shows the median (IQR) PTSD score in a total of women 2(0–17), in emergency cesarean section (EMCS) 17(2–32), and in elective cesarean section (ELCS) 0(0–4).

### 3.3. Risk Factors for PTSD-AC

Univariate logistic regression analyses with the dependent variable as the presence of PTSD (Table 4) showed a significant and compounding association of psychiatric history, number of traumatic events, inclusion in the NICU, and emergency caesarian section with the likelihood of PTSD. Cases with full-term labor, those that breastfed, and those that reported having support from a spouse had a lower likelihood of having PTSD in the univariate analysis. Multiple analyses (Table 4) revealed that the type of caesarian section, inclusion in the NICU, support from a spouse, and breastfeeding were independently associated with the presence of PTSD.

## 4. Discussion

The subject of the present investigation was the identification of risk factors that help the development of PTSD-AC in mothers after EMCS and ELCS, in order to implement appropriate preventive measures in perinatal care. The results show that 40% of women experienced CS as a traumatic childbirth experience (EMCS 71.4%-ELCS 19.6%). Criterion A met 25.6% of all CS (EMCS 54%-ELCS 7.2%) (Table 3). In addition, it was found that PTSD-AC was associated with EMCS, preterm labor, inclusion in the NICU, a lack of breastfeeding and a lack of support from a spouse during the perinatal period.

There are many studies thatindicate the EMCS as a risk factor for the development of postpartum psychological disorders [14,38,39] and postpartum PTSD. For instance, Schwab et al.,shows that all women who had been diagnosed with PTSD had undergone an EMCS (21.15%) [12]. Modaress et al. alsofound high levels of PTSD in women with EMCS (43.8%) in relation to ELCS (23.2%) and those with vaginal delivery [27]. Additionally, Ryding et al. in aninvestigation for PTSD reaction among women with EMCS, showedthat 1/3 of the study population sufferedfrom serious PTSD reactions [21]. On the contrary, the study by Lopez et al. didnot find an association between the kind of CS and PTSD [19]. Additionally, the paper published by van Heumen et al. defends thatpsychosocial characteristics arestronger predictors than the kind of delivery [8]. However, this study found that 13.1% of the study population met the DSM-V criteria for PTSD (31.7% after EMCS and 1% after ELCS), while 8.1% of women were suffering from PTSD Profile according to the DSM –V (14.3% after EMCS and 4.1% after ELCS) (Table 3). The reason for the high difference in PTSD prevalence between the two groups of women is due to urgent surgery.EMCS is an unexpected and more unpleasant birth experience than the ELCS and is also associated with more problems during pregnancy [27,40]. Another reason for this phenomenon might be that the induction of labor takes place before the 41st week of gestation. As a result, this has increased EMCS rates [41].

A remark must be made regarding ours and other findings. This prospective study is the firstto investigate the development of PTSD and PTSD Profile in two groups of high-risk postpartum women (EMCS and ELCS). Furthermore, it is one of the few studieswhere they used all the diagnostic criteria (A, B, C, D, E) for PTSD postpartum, according to the DSM-V(Table 3) [8] and this increases the sensitivity of the measure, compared with similar surveys in the past.

The results of this study showthat preterm labor is associated with PTSD-AC. Only 76.2% of women after EMCS and 87.6% after ELCS had full-term labor and 1/3 of women who met the diagnostic criteria for PTSD or PTSD Profile had preterm labor.A few studies have reported that delivery of a premature infant was a risk factor for the development PTSD and other psychological disorders postpartum [42,43]. Thisstudy agrees with them.One hypothesis is that prematurity is related toemergency situations, inclusion in the NICU and medical complications. Therefore, the risk of losing an infants’ life is real (criterion A) and the cause of the subsequent maternal mental trauma.

Another very important factor predicting PTSD-ACwhich is associated with preterm labor and complications during pregnancy is the inclusion of the infant in the NICU. It is known that maternal distress after the inclusion of the infant in the NICUconsists of a complication of anxiety, depressive and PTSD symptoms and is associated negatively with maternal-infant attachment [44,45]. In our study, inclusion inthe NICU is related to 30.2% of EMCS and 11.3% of ELCS and includes preterm and full-term infants.

For many women, breastfeeding is an extension of the birth experience and it has been proven that it can reduce the trauma of medical delivery.When there is a traumatic childbirth experience, breastfeeding can be disappointing due to traumatic reminders of childbirth. All these feelings can lead to a lack of initiation or premature termination of breastfeeding [46]. In this investigation, the lack of breastfeeding played a determining role in the development of the trauma caused to the mother by the CS, with rates of 95% in the first 24-h nursing mothers without PTSD and 4.5% in nursing mothers with PTSD. These findings are consistent with the research of Hoff et al., which maintains that the lack of breastfeeding influences the development of mother-child attachment, and contributes to the intensity of the distress of the mother and developing postpartum PTSD [47]. Additionally, the maintenance of prolactin at high levels through breastfeeding is an effective way of reducing maternal stress [48].

There was a significant difference between the PTSD-AC and perinatal support from a spouse. In this study, women with poor support from their spouses were more likely to suffer from PTSD or PTSD Profile (28.6%) compared to the women with good support from their spouse (9.8%), the second groupseems to manage better the consequences of traumatic CS.A lot of studies reveal the relationship between social support and good health and suggest that the psychical reaction to stress is under the influence on the level of family and spouse support [49]. Focusing specifically on this, some studies agree with the present study and confirm the effective role ofpartners‘supportto reduce the incidence of postnatal PTSD and PTSD Profile [15,44].

Unfortunately in Greece, there are few supporting services for women exposed to birth trauma For this reason, support from a partner or spouse is one of the main factors protecting from postnatal PTSD.

## 5. Conclusions

The present study identified vulnerability postpartum factors in women who had undergone an emergency or elective cesarean section in a Greek Hospital. Traumatic CS experiences were predictive with the adapted diagnostic measurement tool (PCL-5). The measuresrevised to reflect the new diagnostic criteria and now is one of the few validated measures for PTSD. Women in this study who met all the diagnostic criteria (A, B, C, D, E) of DSM-5 have a provisional diagnosis of PTSD-AC and women who met criteria (B, C, D) only, have a PTSD Profile. From the present study, it has emerged that the risk factors developing PTSD-AC are: EMCS, preterm labor, inclusion inthe NICU, a lack of breastfeeding, and a lack of support from a spouse during the perinatal period. Health professionals who contact women during the perinatal period should inform them so as to ensure the reduction of the above risk factors.Further research should take place to investigate PTSD after birth with EMCS, especially in countries with high percentages of CS, particularly focusing on the differencesin PTSD rates between EMCS and ELCS.

## Figures and Tables

**Table 1:** Sample characteristics in total and according to the type of cesarean section (C-section).

	Total Sample (*N* = 160)	Type of C-Section		*p*
		Emergency (*N* = 63; 39.4%)	Planned (*N* = 97; 60.6%)	
	*N* (%)	*N* (%)	*N* (%)	
Parity				
0	79 (49.4)	46 (73)	33 (34)	<0.001 ^+^
1	61 (38.1)	13 (20.6)	48 (49.5)	
>1	20 (12.5)	4 (6.3)	16 (16.5)	
Type of previous labor				
Vaginal	16 (19.8)	8 (47.1)	8 (12.5)	0.010 ^++^
C-section	62 (76.5)	9 (52.9)	53 (82.8)	
Both	3 (3.7)	0 (0.0)	3 (4.7)	
Psychiatric history	21 (13.1)	8 (12.7)	8 (8.2)	0.337 ^+^
Number of traumatic events, median (IQR)	1 (0–2)	2 (0–4)	1 (0–3)	0.012 ^‡‡^
Medical history	54 (33.8)	23 (36.5)	31 (32)	0.552 ^+^

^+^ Pearson’s chi-square test; ^++^ Fisher’s exact test; ^‡‡^ Mann-Whitney test.

**Table 2:** Characteristics of pregnancy and delivery.

Title	Total Sample (*N* = 160)	Type of C-Section		*p*
		Emergency (*N* = 63; 39.4%)	Planned (*N* = 97; 60.6%)	
	*N* (%)	*N* (%)	*N* (%)	
Conception				
Normal	145 (90.6)	58 (92.1)	87 (89.7)	0.615 ^+^
IVF	15 (9.4)	5 (7.9)	10 (10.3)	
Problems during pregnancy	70 (43.8)	35 (55.6)	35 (36.1)	0.015 ^+^
Gestational week, mean (SD)	37.7 (2.1)	37.4 (3)	38 (1.2)	0.066 ^‡^
Preterm labor	133 (83.1)	48 (76.2)	85 (87.6)	0.059 ^+^
NICU	30 (18.8)	19 (30.2)	11 (11.3)	0.003 _+_
Support from spouse	132 (82.5)	46 (73)	86 (88.7)	0.011 ^+^
Expectations	89 (55.6)	16 (25.4)	73 (75.3)	<0.001 ^+^
Traumatic c-section	64 (40)	45 (71.4)	19 (19.6)	<0.001 ^+^
Breastfeeding	110 (68.8)	36 (57.1)	74 (76.3)	0.011 ^+^

^+^ Pearson’s chi-square test; ^‡^ Student’s *t*-test.

**Table 3:** Criteria and total PTSD in the total sample and according to the type of cesarean section.

	Total Sample (*N* = 160)	Type of C-Section		*p*
		Emergency (*N* = 63; 39.4%)	Planned (*N* = 97; 60.6%)	
	*N* (%)	*N* (%)	*N* (%)	
Criterion Α	41 (25.6)	34 (54)	7 (7.2)	<0.001 ^+^
Criterion B	42 (26.3)	33 (52.4)	9 (9.3)	<0.001 ^+^
Criterion C	42 (26.3)	32 (50.8)	10 (10.3)	<0.001 ^+^
Criterion D	43 (26.9)	33 (52.4)	10 (10.3)	<0.001 ^+^
Criterion E	31 (19.4)	23 (36.5)	8 (8.2)	<0.001 ^+^
Diagnosis				
No PTSD	126 (78.8)	34 (54)	92 (94.8)	<0.001 ^+^
Profile	13 (8.1)	9 (14.3)	4 (4.1)	
PTSD	21 (13.1)	20 (31.7)	1 (1.0)	
PTSD score, median (IQR)	2 (0–17)	17 (2–32)	0 (0–4)	<0.001 ^‡‡^

^+^ Pearson’s chi-square test; ^‡‡^ Mann-Whitney test.

**Table 4:** Results from logistic regression analyses with the dependent variable as the presence of PTSD.

Τotal Sample (N=160)		Diagnosis	Unadjusted OR (95% CI)	*p*		Adjusted OR (95% CI)	*p*
		No PTSD/Profile	PTSD				
		*N* (%)	*N* (%)				
Age		33.3 (5.7)	32 (7.3)	0.97 (0.90–1.04)	0.380	0.89 (0.77–1.03)	0.105
Married/Engaged/In a relationship	No	5 (83.3)	1 (16.7)	1.00^+^		1.00	
	Yes	134 (87.0)	20 (13.0)	0.75 (0.08–6.72)	0.794	0.53 (0.01–54.52)	0.787
Educational level	Primary/Middle/High school graduate	70 (86.4)	11 (13.6)	1.00		1.00	
	University alumni/MSc/PhD	69 (87.3)	10 (12.7)	0.92 (0.37–2.31)	0.863	1.43 (0.33–6.13)	0.630
Financial status	Low	14 (77.8)	4 (22.2)	1.00		1.00	
	Middle/High	125 (88.0)	17 (12.0)	0.48 (0.14–1.61)	0.234	2.26 (0.16–32.05)	0.548
Nationality	Greek	129 (86.6)	20 (13.4)	1.00		1.00	
	Other	10 (90.9)	1 (9.1)	0.65 (0.08–5.31)	0.684	0.16 (0.01–3.11)	0.225
Parity	0	66 (83.5)	13 (16.5)	1.00		1.00	
	≥1	73 (90.1)	8 (9.9)	0.56 (0.22–1.43)	0.222	1.36 (0.11–16.79)	0.810
Previous c-section	No	79 (83.2)	16 (16.8)	1.00		1.00	
	Yes	60 (92.3)	5 (7.7)	0.41 (0.14–1.19)	0.100	0.9 (0.05–15.07)	0.944
Psychiatric history	No	126 (90.6)	13 (9.4)	1.00		1.00	
	Yes	13 (61.9)	8 (38.1)	5.96 (2.09–17.04)	0.001	2.79 (0.55–14.17)	0.216
Number of traumatic events, median (IQR)		1(0–3)	3(1-5)	1.29(1.06–1.57)	0.013	1.34(0.84–2.18)	0.211
Full-term labor	No	18 (66.7)	9 (33.3)	1.00		1.00	
	Yes	121 (91)	12 (9)	0.19 (0.07–0.54)	0.001	0.34 (0.06–1.83)	0.208
Type of c-section	Planned	96 (99.0)	1 (1.0)	1.00		1.00	
	Emergency	43 (68.3)	20 (31.7)	44.65 (5.80–343.50)	<0.001	46.55 (6.00–360.81)	<0.001
NICU	No	121 (93.1)	9 (6.9)	1.00		1.00	
	Yes	18 (60.0)	12 (40)	8.96 (3.31–24.27)	<0.001	9.00 (3.31–24.49)	<0.001
Support from spouse	No	20 (71.4)	8 (28.6)	1.00		1.00	
	Yes	119 (90.2)	13 (9.8)	0.27 (0.10–0.74)	0.011	0.27 (0.10–0.74)	0.011
Expectations	No	50 (70.4)	21 (29.6)				
	Yes	89 (100.0)	0 (0.0)	++	-	-	-
Traumatic c-section	No	96 (100.0)	0 (0.0)				
	Yes	43 (67.2)	21 (32.8)	++	-	-	-
Breastfeeding	No	34 (68.0)	16 (32.0)	1.00		1.00	
	Yes	105 (95.5)	5 (4.5)	0.10 (0.03–0.30)	<0.001	0.08 (0.02–0.25)	<0.001

^+^ indicates reference category ^++^ could not be computed due to no distribution. Note: OR (95% CI) = Odds Ratio (95% Confidence Interval).
